# Rapid Progress in the Use of Immunomodulatory Drugs and Cereblon E3 Ligase Modulators in the Treatment of Multiple Myeloma

**DOI:** 10.3390/cancers13184666

**Published:** 2021-09-17

**Authors:** Grzegorz Charliński, David H. Vesole, Artur Jurczyszyn

**Affiliations:** 1 Department of Hematology, Warmian-Masurian Cancer Center of The Ministry of The Interior and Administration’s Hospital, 10-228 Olsztyn, Poland; grzegorz.charlinski@poliklinika.net; 2 John Theurer Cancer Center at Hackensack Meridian School of Medicine, Hackensack, NJ 07601, USA; david.vesole@hmhn.org; 3 Plasma Cell Dyscrasia Center, Department of Hematology, Faculty of Medicine, Jagiellonian University Medical College, 31-501 Kraków, Poland

**Keywords:** cereblon E3 ligase modulators, immunomodulatory drugs, multiple myeloma, therapy

## Abstract

**Simple Summary:**

Due to the complex mechanism of actions, immunomodulatory drugs (IMiDs) are one of the primary drug classes used to treat multiple myeloma (MM). IMiDs are the backbone of treatment for both newly diagnosed, post-transplant maintenance, and relapsed/refractory MM. The standard of care is a combination of IMiDs, corticosteroids (e.g., dexamethasone) with either a proteasome inhibitor or a monoclonal antibody. Future management will include a quadruplet of all four drug classes. Recent clinical trials have shown that another class of cereblon inhibitors in development, Cereblon E3 ligase modulators (CELMoDs), have significant activity in MM even when refractory to approved IMiDs.

**Abstract:**

Over the past two decades, the improvement in our understanding of the biology of MM and the introduction of new drug classes, including immunomodulatory drugs (IMiDs), proteasome inhibitors (PI), and monoclonal antibodies (MoAb), have significantly improved outcomes. The first IMiD introduced to treat MM was thalidomide. The side effects observed during treatment with thalidomide initiated work on the synthesis of IMiD analogs. Subsequently, lenalidomide and pomalidomide were developed, both with different safety profiles, and they have better tolerability than thalidomide. In 2010, the cereblon (CRBN) protein was discovered as a direct target of IMiDs. By binding to CRBN, IMiDs change the substrate specificity of the CRBN E3 ubiquitin ligase complex, which results in the breakdown of internal Ikaros and Aiolos proteins. Most clinical trials conducted, both in newly diagnosed, post-transplant maintenance and relapsed/refractory MM, report a beneficial effect of IMiDs on the extension of progression-free survival and overall survival in patients with MM. Due to side effects, thalidomide is used less frequently. Currently, lenalidomide is used at every phase of MM treatment. Lenalidomide is used in conjunction with other agents such as PIs and MoAb as induction and relapsed therapy. Pomalidomide is currently used to treat relapsed/refractory MM, also with PIs and monoclonal antibodies. Current clinical trials are evaluating the efficacy of IMiD derivatives, the CRBN E3 ligase modulators (CELMoDs). This review focuses on the impact of IMiDs for the treatment of MM.

## 1. Introduction

Multiple myeloma (MM) is a clonal plasma cell (PC) malignancy still considered incurable with current treatments. MM is manifested as an uncontrolled expansion of malignant PCs in the bone marrow, almost always corresponding with the production of a monoclonal (M) protein in the serum and/or urine. MM accounts for 1.8% of all malignancies and 10–15% of hematologic malignancies [1]. Until the end of the 20th century, the standard induction therapy for MM consisted of corticosteroids alone, melphalan/prednisone, or the combination of vincristine, doxorubicin, and dexamethasone (VAD). High-dose melphalan with autologous stem cell transplantation (ASCT) was employed as consolidation for transplant-eligible patients after induction therapy. The median overall survival (OS) of patients with MM at this time was only 2–3 years. Subsequently, the introduction of immunomodulatory drugs (IMiDs), proteasome inhibitors (PIs), and monoclonal antibodies (MoAbs) have improved treatment outcomes and extended the median OS 5–15+ years depending on the stage of the disease and genetic abnormalities [2,3,4].

Immunomodulatory drugs are oral drugs used to treat MM and have unique mechanisms of action, including anti-cancer and anti-inflammatory effects, and affect the human immune system [5]. The introduction of IMiDs presenting a pleiotropic mechanism of action fits well with the current approach to anti-myeloma therapy, which has a triple effect: inducing direct apoptosis against malignant tumor cells, interfering with the interaction of the tumor with bone marrow stromal cells, and the increase in the anti-tumor immune response [6].

Despite the similarity in chemical structure, IMiDs differ in their side effect profile and, interestingly, show only moderate cross-reactivity, allowing for sequential treatment. For this reason, these drugs are used at all stages of the treatment of MM.

Currently, these drugs are considered standard of care for induction therapy for transplant-eligible and transplant-ineligible patients, maintenance therapy after ASCT, and treatment of relapsed/refractory MM (RRMM).

Thalidomide (α-N-phthalimido-glutarimide) is a derivative of glutamic acid and was synthesized in 1954. Initially, it was used as a sedative and barbiturate antiemetic. In 1957, thalidomide was approved for treatment in the first trimester for pregnancy-associated nausea, but unfortunately, it was associated with birth defects (phocomelia) as cereblon (CRBN) receptors are present in the limbs of developing fetuses [7,8,9]. Thalidomide was later used in inflammatory diseases such as leprosy (erythema nodosum leprosum) and Behçet’s syndrome. Subsequently, thalidomide, due to its anti-angiogenic activity, was postulated to be effective for the treatment of MM disease with extensive bone marrow angiogenesis, and it was proven effective in MM due to its anti-angiogenic and immunomodulatory effects [10]. More than 20 years have passed since the initial publication describing the effectiveness of thalidomide in the treatment of MM [11].

As thalidomide is not a cytotoxic agent and has potential in vitro synergy with other drugs, including dexamethasone, many combinations of thalidomide have been developed for the treatment of MM. The side effects (teratogenic and sedative effects and development of peripheral neuropathy) observed during treatment with thalidomide promoted the development of thalidomide analogs with more significant immunomodulatory activity and better safety profile [12]. As a result, a chemical structure modification was undertaken that led to the development of lenalidomide and pomalidomide.

Lenalidomide (CC-5013) is the 4-amino-glutamyl analogue of thalidomide. Unlike thalidomide, lenalidomide is not sedative, and the incidence of sensory axonal neuropathy is less frequent, but still possible [13,14].

In the United States, in 2006, the Food and Drug Administration (FDA) approved lenalidomide in combination with dexamethasone (Rd) for the treatment of RRMM. In 2015, lenalidomide was approved for the treatment of newly diagnosed MM (NDMM). Between 2015 and 2019, five triple-drug regimens containing lenalidomide were approved for the treatment of RRMM: bortezomib/lenalidomide/dexamethasone (VRd), carfilzomib/lenalidomide/dexamethasone (KRd), ixazomib/lenalidomide/dexamethasone (Ixa-Rd), elotuzumab/lenalidomide/dexamethasone (Elo-Rd), and daratumumab/lenalidomide/dexamethasone (Dara-Rd). In 2017, lenalidomide was approved for maintenance therapy after ASCT.

Pomalidomide (CC-4047) is the 4-amino-2- (2,6-dioxopiperidin-3-yl) isoindole-1,3-dione which has direct antiproliferative, pro-apoptotic, and anti-angiogenic effects. It has a modulating effect on bone resorption and the immune system [15]. The United States FDA approved pomalidomide in 2013 for the treatment of patients with RRMM who had received at least two prior therapies, including lenalidomide and bortezomib. Currently, the FDA has approved pomalidomide for the treatment of RRMM in combination with dexamethasone (Pd) and Pd in combination with isatuximab (Isa-Pd), daratumumab (Dara-Pd), and elotuzumab (Elo-Pd).

A new group of thalidomide analogs are the CRBN E3 ligase modulators (CELMoDs), which leads to the degradation of Ikaros and Aiolos [16].

**Figure 1:**
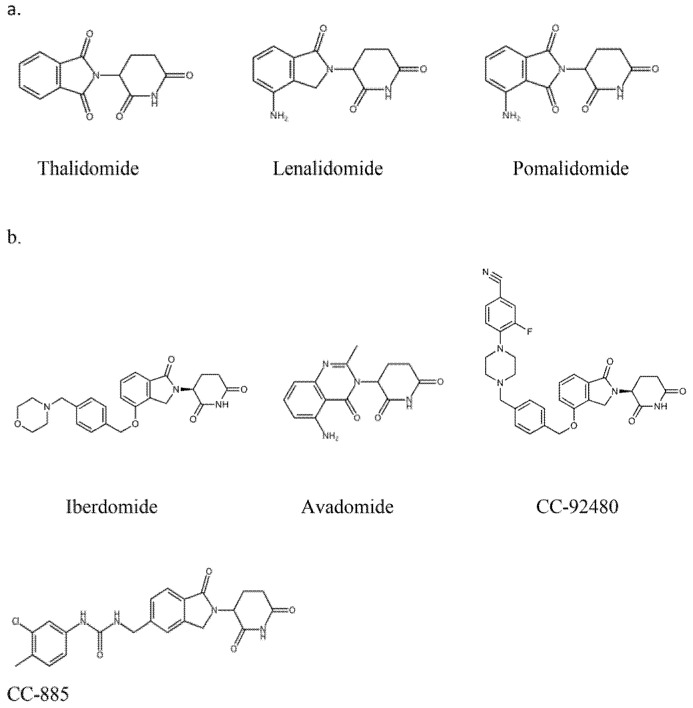
Chemical structure of immunomodulatory drugs (**a**) and Cereblon E3 ligase modulators (**b**).

Talking into account chemical structure, both IMiDs and CELMoDs contain glutarimide rings and isoindolinone rings. In the chemical structure of CELMoDs, there are phenyl and morpholino moieties that allow interaction with CRBN. [17,18]. The chemical structure of the drugs IMiDs and CELMoDs is shown in Figure 1. The family of CELMoDs include iberdomide, avadomide, CC-92480, and CC-885.

## 2. Mechanism of Action of Immunomodulatory Drugs and Cereblon E3 Ligase Modulators in the Treatment of Multiple Myeloma

The IMiDs and CELMoDs share common, but at the same time, slightly different, mechanisms of action leading to differentiated cellular effects. These differences arise from the presence, amount, and preference for essential substrate proteins, including transcription factors.

The mechanism of action of IMiDs in MM cells was initially thought as an anti-angiogenesis process [19]. Subsequently, thalidomide and its analogs were found to exert direct and indirect anti-tumor activity through immunomodulation. Lenalidomide and pomalidomide show more significant immediate anti-tumor effect than thalidomide in vitro [20,21]. Additionally, these drugs modulate the interaction of MM cells with their microenvironment [22].

In 2010, the anti-myeloma activity of IMiDs was discovered to work through the inhibition of CRBN, a protein that dictates the substrate specificity of the CRL4CRBN E3 ubiquitin ligase [9,23,24].

**Figure 2:**
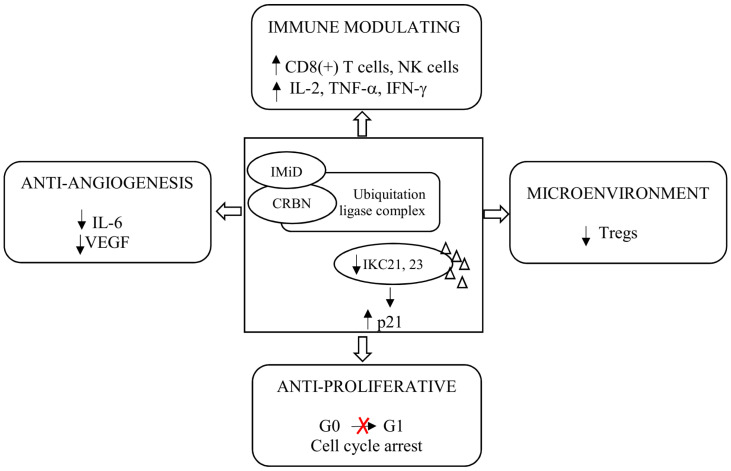
Mechanism of action of immunomodulatory drugs. Abbreviations: CRBN: Cereblon; IFN-γ: Interferon γ; IL: Interleukin; IMiD: Immunomodulatory drug; NK: Natural killer; Tregs: Regulatory T cells; TNF-α: Tumor Necrosis Factor α; and VEGF: Vascular Endothelial Growth Factor.

The IMiDs, by binding CRL4CRBN E3 ligase, cause ubiquitination and degradation of disease-related proteins. The components of the CRL4CRBN E3 ligase and its activity are important for the anti-myeloma activity of IMiDs [25]. The key neosubstrates in PCs are Ikaros (IKZF1) and Aiolos (IKZF3) [26,27]. These transcription factors (TFs) regulate cell fate in normal lymphopoiesis and PCs development [28]. Both Ikaros and Aiolos increase interferon regulatory factor 4 (IRF4) and c-MYC, which form a positive autoregulatory loop that is necessary for PCs’ proliferation. These four TFs are called the axis of Ikaros. The IMiDs lead to the rapid degradation of Ikaros and Aiolos through CRBN-dependent ubiquitination, leading to the downregulation of IRF4 and c-MYC [29]. As mentioned above, IMiDs, in addition to their direct anti-myeloma effect, have an indirect anti-myeloma effect, reducing the secretion of pro-inflammatory cytokines, including tumor necrosis factor-alpha (TNF-α) interleukin (IL)-1, IL-6, 12, and IL-16. The consequence of the reduction in these cytokines is the inhibition of the proliferation and migration of neoplastic PCs and apoptosis [30]. Lenalidomide and pomalidomide, compared to thalidomide, more strongly induce apoptosis of neoplastic PCs by activating tumor suppressor genes, including p21, an inhibitor of cyclin-dependent kinase (CDK). Inhibition of CDK activity arrests the cell cycle in the G0/G1 phase and apoptosis of the PC [22]. IMiDs enhance co-stimulation of T lymphocytes, which leads to increased secretion of interferon γ (IFN-γ) and IL-2, a proliferation of clonal T lymphocytes, and activation of NK lymphocytes [31]. In preclinical studies, lenalidomide and pomalidomide were 300–1200 times more potent than thalidomide at co-stimulating T cells [32,33]. Both lenalidomide and pomalidomide increase the action of NK cells in destroying PC. Lenalidomide further activates NKT cells [34,35]. The main mechanisms of action of IMiDs are presented in Figure 2.

Cereblon E3 ligase modulators, compared to IMiDs, are characterized by a higher affinity for CRBN and cause a stronger degradation of Ikaros and Aiolos, which is associated with a stronger anti-myeloma and immunomodulatory effect [36,37]. This is the most important difference between these groups of drugs. The potency of CELMoDs may explain their more significant activity at lower levels of CRBN, or in cases of resistance to IMiDs, due to mutations in CRBN.

**Figure 3:**
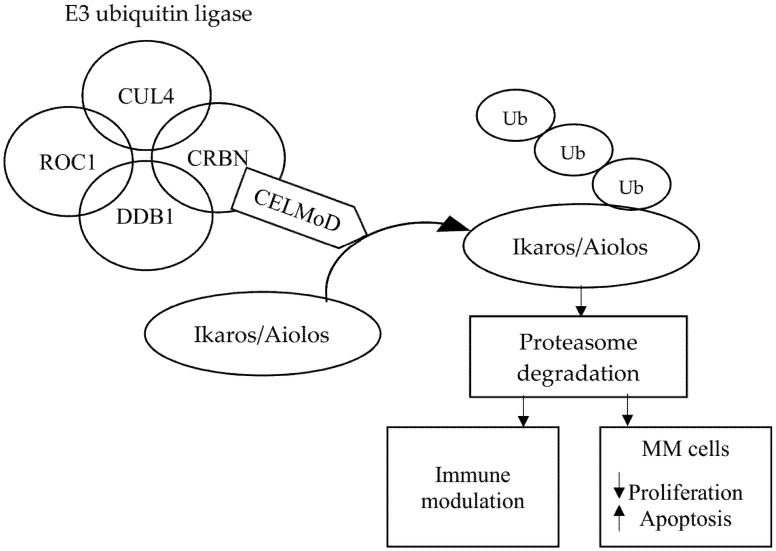
Molecular mechanism of action of cereblon E3 ligase modulators in multiple myeloma. Abbreviations: CELMoD: Cereblon E3 ligase modulator; CRBN: Cereblon; CUL4: Cullin-4; DDB1: Damaged DNA binding protein 1; MM: Multiple myeloma; ROC1: Regulator of cullins 1; and Ub: Ubiquitination.

CELMoDs bind specifically to CRBN, thus influencing the activity of E3 ubiquitin ligase and targeting specific substrate proteins, causing them to ubiquitinate. This action degrades some transcription factors, which are proteasome-mediated transcriptional repressors. The consequence of this action is immunomodulation, including the activation of T lymphocytes and the degradation of proteins that play an important role in the proliferation of cancer cells. The mechanism of action of CRL4CRBN E3 ubiquitin ligase and its effects through CRBN-based small molecules are presented in Figure 3.

These CELMoDs, Iberdomide and CC-92480, have 10–20 times greater affinity for CRBN and degrade Ikaros and Aiolos more strongly than lenalidomide and pomalidomide [36,37].

## 3. Immunomodulatory Drugs in Newly Diagnosed Multiple Myeloma

Decisions regarding the treatment of NDMM depend on age, performance status, comorbidities, and patient and physician preferences [38]. In Europe, induction therapy followed by ASCT is used for the first-line treatment for patients up to 65–70 years of age; in the United States, there is no specific upper age limit for consideration of ASCT. Patients who are not eligible for ASCT are treated with standard doses of drugs [38].

In the first-line treatment, thalidomide and lenalidomide are used in transplant eligible and ineligible patients.

### 3.1. Thalidomide

#### 3.1.1. Thalidomide for the Treatment of Newly Diagnosed Multiple Myeloma in Patients Eligible for ASCT

The introduction of thalidomide improved the treatment outcomes of patients with MM. Thalidomide in combination with bortezomib and dexamethasone (VTD) is a standard induction regimen in transplant-eligible patients with NDMM. A number of randomized clinical trials have confirmed the superior efficacy of VTD over other drug combinations used in pre-ASCT induction treatment [39,40].

In a phase 2 trial, total therapy 3 (TT3), Barlogie et al. used VTD in combination with cisplatin/doxorubicin/cyclophosphamide/etoposide (VTD-PACE) as induction before, and consolidation after, ASCT. After 24 months, 83% of patients achieved near-complete response (nCR). The estimated 2-year event-free survival and OS were 84% and 86%, respectively [41].

Of particular importance are the results of the phase 3 study by Cavo et al., which compared VTD versus thalidomide and dexamethasone (TD) used in induction before ASCT. After induction treatment, a complete response (CR) or nCR was achieved by 33.1% and 13.7%, respectively (*p* < 0.0001), while the 3-year progression-free survival (PFS) was significantly longer in the VTD group (60% vs. 48%, respectively; *p* = 0.042) [39]. In addition, a randomized phase 3 trial by the Intergroupe Francophone du Myelome (IFM) compared VTD with bortezomib, cyclophosphamide, and dexamethasone (VCD) as pre-ASCT induction therapy, and demonstrated an overall response rate (ORR) of 92.3% vs. 83.4% (*p* = 0.01), respectively, and ≥ VGPR was 66.3% vs. 56.2%, respectively (*p* = 0.05) [40]. 

In 2019, the CASSIOPEIA trial, a randomized phase 3 trial comparing the quadruplet consisting of daratumumab (a human anti-CD38 monoclonal antibody) with VTD (Dara-VTD) with the triplet VTD demonstrated a CR or better in 39% vs. 26%, respectively (*p* < 0.0001) [42]. The results of the CASSIOPEIA study contributed to the Dara-VTD protocol being the recommended induction treatment for ASCT-eligible patients in Europe [43].

The results of selected studies evaluating the use of thalidomide in the treatment of NDMM patients eligible for ASCT are summarized in Table 1.

#### 3.1.2. Thalidomide for the Treatment of Newly Diagnosed Multiple Myeloma in Patients Ineligible for ASCT

The efficacy of thalidomide in the treatment of transplant-ineligible patients with NDMM has been assessed in six randomized clinical trials comparing the then standard of care combination of melphalan and prednisone (MP) with the triplet of MP and thalidomide (MPT) [45,46,53,54,55,56]. All of the studies demonstrated that the MPT group had a significant improvement in PFS, and four studies reported an improvement in OS. In a meta-analysis of 1685 patients randomized to these studies, the addition of thalidomide to MP protocol had a significant effect on OS (HR, 0.83; *p* = 0.004). In the studied groups, MPT showed better PFS (HR, 0.68; *p* < 0.0001), and OS was 32.7 months vs. 39.3 months, respectively [57]. The use of MPT has been replaced by other more effective and less toxic treatments.

A randomized phase 3 study by GIMEMA evaluated the efficacy of a four-drug combination of VMP plus thalidomide (VMPT) followed by bortezomib/thalidomide maintenance treatment (VMPT-VT) compared to VMP alone in NDMM patients not eligible for ASCT; the PFS was significantly better in the VMPT-VT group [58].

The results of selected studies evaluating the use of thalidomide in the treatment of NDMM patients ineligible for ASCT are summarized in Table 1.

### 3.2. Lenalidomide

#### 3.2.1. Lenalidomide for the Treatment of Newly Diagnosed Multiple Myeloma in Patients Eligible for ASCT

The incorporation of lenalidomide and dexamethasone as induction therapy in transplant-eligible NDMM have shown response rates between 68–91% [47,59]. Ultimately, the efficacy of Rd led to the combination with PI, bortezomib (VRD), one of the most frequently used induction treatments for patients with NDMM. In phase 3 trials, VRD has resulted in CR ranging from 23–33% of patients [60,61,62]. A direct comparison of Rd versus VRd was evaluated in the randomized phase 3 SWOG S0777 clinical trial [48]. VRd was statistically superior in regard to PFS, 41 months versus 29 months, respectively (*p* = 0.003), and the median OS, NR vs. 69 months, respectively (*p* = 0.011) [48]. 

Currently, VRd is now considered the gold standard for induction treatment in the US and many countries outside of Europe [60,63,64]. Of note, the European Medicines Agency (EMA) does not approve VRD for induction treatment pre-ASCT.

Carfilzomib, the second-generation PI, has also been evaluated in combination with Rd (KRd) as induction therapy for NDMM, either with twice weekly or once weekly carfilzomib [44,65,66]. In a phase 2 study, after using four cycles of KRd, before ASCT, the ORR was 97%, including a CR of 16%; at 60 months, the PFS was 72%, and the OS was 84% [66]. Due to high ORR in phase 2 trials, a head-to-head phase 3 trial of VRD versus KRD in nontransplant eligible patients was completed (ENDURANCE) [44]. There were no statistically significant differences in PFS and OS in this study; however, the depth of response and rapidity of response favored the KRd arm. Peripheral neuropathy was higher in the VRd arm, whereas cardiac/pulmonary/renal toxicities were higher in the KRd arm. The GRIFFIN trial, a randomized phase 2 trial compared VRD to daratumumab/VRD (Dara-VRD) demonstrated a 24 months PFS of 89.8% vs. 95.8% (*p* = NS) [67]. In nonrandomized clinical MANHATTAN trial, daratumumab/KRd (Dara-KRd, carfilzomib weekly) 100% of patients achieved ≥ VGPR, and 71% patients were MRD-negative. After 12 months, PFS and the OS rates were 98% and 100%, respectively [68]. The phase 2 FORTE trial compared KRd with followed ASCT (KRd_ASCT), 12 cycles of KRd(12), and carfilzomib, cyclophosphamide, and dexamethasone (KCd) with followed ASCT (KCd_ASCT). In the final analysis, the median PFS in the analyzed groups was NR, 57 months, 53 months, respectively (KRd_ASCT vs. KCd_ASCT: HR, 0.53; *p* < 0.001; KRd_ASCT vs. KRd12: HR, 0.64; *p* = 0.023; KRd12 vs. KCd_ASCT: HR, 0.82; *p* = 0.262). The 3-year OS was 90% with KRd_ASCT and KRd12 vs. 83% with KCd_ASCT [69].

The results of selected studies evaluating the use of lenalidomide in the treatment of NDMM patients eligible for ASCT are summarized in Table 1.

#### 3.2.2. Lenalidomide in the Treatment of Newly Diagnosed Multiple Myeloma in Patients Ineligible for ASCT

The FIRST trial was a randomized three-arm study in transplant-ineligible NDMM patients: Rd (continuation until progression), Rd-18 (18 cycles), and MPT. Median PFS for the three groups was 26, 21, and 21.9 months, respectively [70]. When comparing the continuous Rd arm with the MPT arm, an improvement in the 4-year OS rate was observed. There was no difference in Rd-18 versus MPT. In the final analysis, the median OS in the analyzed groups was 59.1 months, 62.3 months, and 49.1 months, respectively (Rd(cont) vs. MPT: HR, 0.78; *p* = 0.0023) [49].

A direct comparison of melphalan, prednisone, and lenalidomide (MPR) followed by lenalidomide maintenance (MPR-R) versus MPR versus MP was evaluated in the randomized MM-015 phase 3 clinical trial [50]. The median PFS was significantly longer with MPR-R (31 months) than with MPR (14 months; HR, 0.49; *p* < 0.001) or MP (13 months; HR, 0.40; *p* < 0.001).

Two randomized phase 3 trials, ECOG E1A06 and HOVON87/NMSG18, have compared MPT treatment with melphalan/prednisone/lenalidomide (MPR) in NDMM patients ineligible for ASCT. In ECOG, E1A06 administered thalidomide or lenalidomide maintenance treatment after 12 cycles of either MPT (MPT-T) or MPR (MPR-R) [51]. There were no statistically significant differences in response rates and medians of PFS and OS. In the HOVON study, after nine cycles of induction with either MPT or MPR was followed by maintenance treatment with thalidomide or lenalidomide [71]. As in ECOG E1A06, there were no significant differences in ORR, PFS, and OS. More recently, the phase 3 MAIA study compared Rd vs. Dara-Rd in first-line treatment in transplant-ineligible patients [72]. In the study update, PFS was superior with Dara-Rd (median PFS: 34 vs. NR, respectively (HR, 0.54; *p* < 0.001) [52].

The results of selected studies evaluating the use of lenalidomide in the treatment of NDMM patients ineligible for ASCT are summarized in Table 1.

## 4. Immunomodulatory Drugs in Maintenance Therapy after ASCT

### 4.1. Thalidomide

Thalidomide maintenance treatment has been studied in a number of phase 3 trials [73,74,75] and meta-analyses [76,77]. All of the reported trials showed that thalidomide improved PFS, but there was no apparent effect on the improvement of OS. The International Myeloma Working Group (IMWG) meta-analysis has shown that thalidomide maintenance therapy reduces the risk of progression or death by 35% [78]. The side effects that lead to treatment discontinuation are a limiting factor in using thalidomide for maintenance therapy [73,74,75,76]. In addition, the Myeloma IX trial found that, in high cytogenetic risk, thalidomide had a negative impact on outcomes [78].

### 4.2. Lenalidomide

Currently, lenalidomide is considered the standard of care for maintenance therapy after ASCT. Phase 3 randomized trials comparing maintenance lenalidomide to observation all demonstrated a beneficial effect of lenalidomide in prolonging PFS. However, only one of these trials showed an OS benefit (and not a primary endpoint). In the meta-analysis of the CALGB, IFM, and GIMEMA, Myeloma IX found that lenalidomide used in maintenance therapy significantly improved OS even though only one of these trials showed an OS benefit independently [79]. The value of lenalidomide used as monotherapy as maintenance therapy has been demonstrated in other pivotal studies [80]. In IFM 2009, lenalidomide maintenance treatment for one year after VRd plus ASCT induction vs. prolonged VRd increased the incidence ≥ VGPR (78% vs. 69% to 85% vs. 76%, respectively) [81]. Similarly, the ongoing phase 2 trial lenalidomide-elotuzumab as maintenance treatment after ASCT showed an improved response in 33% of patients, with 20% conversion to CR [82]. The randomized studies of Myeloma XI, EMN02/HO95, and RV-MM-EMN-441 demonstrated significantly higher conversion rates from MRD-positive to MRD-negative status of approximately 27–48% lenalidomide maintenance therapy [79,83,84,85].

Lenalidomide in maintenance therapy after ASCT is considered a standard of care [43].

## 5. Immunomodulatory Drugs in the Treatment of Relapsed/Refractory Multiple Myeloma

### 5.1. Thalidomide

Thalidomide is currently rarely used for the treatment of RRMM. In the initial phase 2 study, thalidomide, as a single agent, at doses ranging from 50 mg to 800 mg/day, resulted in an ORR of approximately 30%, including a CR in 16% of patients [86]. In a phase 3 study, Kropff et al. compared treatment with thalidomide 100 mg, 200 mg, and 400 mg/day with dexamethasone [87]. The median time to progression was 6.1 months (thalidomide 100 mg/day), 7.0 months (thalidomide 200 mg/day), 7.6 months (thalidomide 400 mg/day) and 9.1 months (dexamethasone), respectively. ORR and OS were similar in all groups. In contrast, the median duration of response was significantly longer in the thalidomide groups [85].

Thalidomide, in combination with dexamethasone, induces ORR in 41–65% of RRMM patients. The most frequently reported AEs were constipation, somnolence, peripheral polyneuropathy, and thrombosis [88,89].

### 5.2. Lenalidomide

The current ESMO guidelines recommend Rd or Rd-based triplet (e.g., Dara-Rd, KRd, Ixa-Rd, or Elo-Rd) in two patient groups: lenalidomide sensitive patients in the first line, and patients sensitive and refractory to bortezomib in the first-line therapy [43]. Although there is no official definition of early versus late MM relapses, it is currently recommended to use lenalidomide in early relapses (as second-line therapy).

The phase 3 ASPIRE study comparing KRd with Rd reported significantly prolonged PFS in the KRd arm (median PFS: 26.3 months vs. 17.6 months, respectively; *p* = 0.0001). In the most recent update, KRd was shown to also OS (median OS: 48.3 months vs. 40.4 months, respectively, *p* = 0.0045) [90]. In the final analysis of the phase 3 TOURMALINE-MM1 trial comparing Ixa-Rd to Rd, the median OS in the analyzed groups was 53.5 months and 51.6 months, respectively (HR, 0.94; *p* = 0.495). Treatment with Ixa-Rd demonstrated a benefit in subgroups, including patients with del(17p) (HR, 0.916), high-risk cytogenetics (HR, 0.870), and expanded high-risk cytogenetics (HR, 0.862). [91].

In the most recent update of the phase 3 POLLUX study comparing Dara-Rd with Rd, Dara-Rd significantly prolonged PFS (median PFS: 44.5 months vs. 17.5 months, respectively, HR, 0.44; *p* < 0.0001). ORR was 92.9% vs. 76.4%, respectively (*p* < 0.001) and MRD negativity was 30.4% vs. 5.3%, respectively (*p* < 0.0001) [92]. Finally, in the phase 3 ELOQUENT-2 study comparing Elo-Rd to Rd, Elo-Rd reduced the risk of disease progression or death by 27% (median PFS 18.5 months vs.14.9 months, respectively, *p* = 0.0014) [93].

The results of selected studies evaluating the use of lenalidomide in the treatment of RRMM are summarized in Table 2.

### 5.3. Pomalidomide

Pomalidomide is used to treat RRMM both in combination with dexamethasone (Pd) and in Pd-based triple protocols, e.g., Dara-Pd, Isa-Pd, elotuzumab (Elo-Pd), carfilzomib (KPd), bortezomib (PVd). In the EHA-ESMO guidelines, pomalidomide is recommended for use in the second-line therapy in combination with bortezomib and dexamethasone to treat patients sensitive to bortezomib, as well as used in the first line for those sensitive and resistant to lenalidomide. In the third line of treatment, it is recommended to use pomalidomide in triples based on Pd, in combination with, e.g., cyclophosphamide, isatuximab, daratumumab, or elotuzumab [43].

A number of phase 3 trials have shown the efficacy of pomalidomide in RRMM. The NIMBUS study compared Pd with high dose dexamethasone in patients with RRMM: the median PFS and OS were statistically longer in the group of patients treated with Pd [94,100]. In the STRATUS trial, 32.6% of patients achieved at least partial response (PR) after treatment with Pd, the median PFS was 4.6 months, and the median OS was 11.9 months [101]. Further studies looked at the efficacy and safety of Pd in combination with a third anti-MM drug. Pomalidomide, in combination with dexamethasone, induces ORR in about 30% of RRMM patients with a median PFS of about 4.5 months, while adding a third drug to Pd increases the ORR to 50–85% with the median PFS of 9.5–12.5 months [94,95,96,97,98,99,100,101,102,103,104,105,106].

In a phase 1/2 study, Larocca et al. in the protocol, pomalidomide in combination with cyclophosphamide and prednisone defined a maximum tolerated dose of pomalidomide of 2.5 mg/day with an ORR of 51% [102]. Baz et al. studied pomalidomide in combination with cyclophosphamide and dexamethasone (PCD). ORR was 64.7%, with a median PFS of 9.5 months [95]. The use of the PCD regimen in the first relapse of MM treated with the VRd protocol in patients qualified for ASCT, after four treatment cycles, 85% of patients achieved at least PR, and this treatment may be a bridge for salvage ASCT [103].

In the randomized phase 3 OPTIMISMM trial, pomalidomide, bortezomib, and dexamethasone (PVd) was superior to bortezomib and dexamethasone (Vd). The median PFS was 11.2 vs. 7.1 months, respectively (*p* < 0.0001), and ORR was 82.2% vs. 50.0%, respectively (*p* < 0.001) [96,104]. The use of Pd in combination with other PIs, carfilzomib (KPd) or ixazomib (Ixa-Pd) are also effective methods of treatment for heavily treated RRMM patients. The overall response rate was 50% and 48%, respectively, and the median PFS was 7.2 and 8.6 months, respectively. [105,106].

Adding an anti-CD38 monoclonal antibody (MoAb) has been studied in combination with Pd in phase 3 trials: APOLLO trial compared daratumumab plus Pd (Dara-Pd) versus Pd; the median PFS was 12.4 months vs. 6.9 months, respectively (*p* = 0.0018). Overall response rate was 69% vs. 46%, with VGPR or better in 51% vs. 20% (*p* < 0.0001) [97]. The ICARIA-MM study compared Isa-Pd versus Pd; Isa-Pd reduced the risk of disease progression and death by 40%; the median PFS was 11.5 months vs. 6.5 months, respectively (*p* = 0.001) and ORR was 60.4% vs. 35.3%, respectively (*p* < 0.0001) [98]. Finally, the anti-SLAMF7 MoAb, elotuzumab was studied in the phase 3 ELOQUENT-3 study elotuzumab to Pd (Elo-Pd) versus Pd; the median PFS was 10.3 months vs. 4.7 months [99].

The results of selected studies evaluating the use of pomalidomide in the treatment of RRMM are summarized in Table 2.

## 6. The New Generation of Immunomodulatory Drugs—Cereblon E3 Ligase Modulators

Cereblon E3 ligase modulators (CELMoDs) are analogs of thalidomide. The family of CELMoDs in clinical trials include iberdomide (CC-220), avadomide (CC-122), CC-92480, and CC-885.

### 6.1. Iberdomide (CC-220)

In preclinical studies, iberdomide in combination with bortezomib has been shown to cause more significant degradation of Ikaros and Aiolos and more profound apoptosis than the other IMiDs used in combination with bortezomib. Iberdomide synergistically increases the activity of daratumumab [107]. In a phase 1 study in healthy volunteers, 6 mg daily was considered a safe dose of iberdomide [108]. The safety, tolerability, pharmacokinetics, and initial efficacy of increasing the dose of iberdomide alone or in combination with dexamethasone, with and without daratumumab in RRMM are being assessed in an ongoing phase 1/2 clinical trials. Based on preliminary results from a study with an ascending dose of 0.3 to 1.3 mg of iberdomide in combination with dexamethasone, the ORR was 29%, and the clinical benefit rate was 45%. The most frequently reported severe AEs were neutropenia (29%), infections (25%), and thrombocytopenia, generally lower than that observed with lenalidomide or pomalidomide (12%) [109].

In the phase 1/2, CC-220-MM-001 trial compared iberdomide in combination with dexamethasone and daratumumab (Iber-Dd) or bortezomib (Iber-Vd) in RRMM: the Iber-Dd cohort ORR was 35%, and the Iber-Vd cohort ORR was 50%. Most frequent grade 3–4 treatment-emergent AEs were neutropenia (50%), leukopenia (22%), and anemia (22%) with Iber-Dd; and neutropenia (20%) and thrombocytopenia (20%) with Iber-Vd [110].

In another phase 1/2 study, Lonial et al. investigated the safety and efficacy of Iber-Dd, Iber-Vd, and iberdomide in combination with carfilzomib and dexamethasone (Iber-Kd) in RRMM: the ORR was 41%, 58%, and 57%, respectively [111]. The most frequently observed AEs in the Iber-Dd group were: neutropenia (63% of patients), leukopenia (28%), and anemia (28%); in the Iber-Vd group: neutropenia (29%) and thrombocytopenia (25%); and in Iber-Kd group: neutropenia (43%).

### 6.2. Avadomide (CC-122)

Avadomide is a CELMoD that has a conserved glutarimide in its structure to bind CRBN. Avadomide is currently being investigated in non-Hodgkin’s lymphoma, MM, and chronic lymphocytic leukemia/small lymphocytic lymphoma. In a phase 1 study, the maximum tolerated dose (MTD) of avadomide is 3.0 mg/day on the 28-day program. The most common AEs were fatigue (44%), neutropenia (29%), and diarrhea (15%). In the study group, two patients had MM. One of them achieved disease stabilization [112].

### 6.3. CC-92480

In a phase 1 dose-escalation study of CC-92480 (NCT03374085) in heavily treated RRMM (median prior lines of therapy: 6), the ORR was 21%, and the efficacy was dose- and schedule-dependent. For the two 1.0 mg QD regimens (10/14 days and 21/28 days), the response was 48% independent of resistance to prior immunomodulators [113]. The most common grade 3 and 4 AEs were neutropenia (53%), infections (30%), anemia (29%), thrombocytopenia (17%), and with grade 3 fatigue (9%).

## 7. Conclusions

Immunomodulatory drugs are a class of drugs that are used at every phase of MM treatment. The complex mechanism of action of IMiDs produces a synergistic effect, increasing the effectiveness of other drugs used to treat MM. Thalidomide has been used to treat MM for over 20 years, and it is still part of many treatment regimens. Lenalidomide is currently the most widely used IMiDs in the treatment of MM for newly diagnosed, maintenance therapy, and RRMM. Due to the increasing number of patients with lenalidomide-refractory MM, pomalidomide is considered essential in this patient groups. The discovery of CRBN aided understanding of the mechanism of action of IMiDs, and led to the development of a new class of IMiDs known as CELMoDs. The IMiDs are very small molecules with little affinity for CRBN and no measurable affinity for the target proteins Ikaros and Aiolos, so the direct and high-affinity interaction between substrate protein and the ligand is unlikely to the observed ubiquitination and degradation [114]. In turn, CELMoDs show a high affinity to CRBN, which leads to the degradation of Ikaros and Aiolos [36]. Based on current knowledge, it appears that these drugs may play an essential role in the treatment of MM in the future, including overcoming treatment refractory, and may provide the basis for new treatment options.

## Figures and Tables

**Table 1:** Results of randomized studies in newly diagnosed multiple myeloma.

Trial/Author	Regimen	Patients, *n*	ORR, %	≥VGPR, %	Median PFS, mo or %	Median OS, mo or %
Transplant eligible						
Thalidomide-based						
Cavo et al. [39]	TD	238	87	31	NR; 60% at 36 mo	NR; 88% at 36 mo
	VTD	236	96	62	NR; 48% at 36 mo	NR; 90% at 36 mo
Moreau et al. [40]	VCD	169	83	56	NR	NR
	VTD	169	92	66	NR	NR
CASSIOPEIA [42]	Dara-VTD	543	92	83	NR; 93% at 18 mo	NR
	VTD	542	90	78	NR; 85% at 18 mo	NR
Lenalidomide-based						
ENDURANCE [44]	VRd	542	84	65	34.4	NR; 84% at 36 mo
	KRd	545	87	74	34.6	NR; 86% at 36 mo
Transplant ineligible						
Thalidomide-based						
Palumbo et al. [45]	MPT	167	69	45	21.8	45.0
	MP	164	48	15	14.5	47.6
Palumbo et al. [46]	VMPT-VT	254	89	59	35.3	NR; 61% at 60 mo
	VMP	257	81	50	24.8	NR; 51% at 60 mo
Lenalidomide-based						
Rajkumar et al. [47]	RD	223	79	NR	NR	NR; 75% at 24 mo
	Rd	225	68	NR	NR	NR; 87% at 24 mo
SWOG S0777 [48]	Rd	261	79	53	29	69
	VRd	264	90	75	41	NR; 69% at 60 mo
FIRST [49]	Rd (cont)	535	81	48	26	59
	Rd18	541	79	47	21	62
	MPT	547	67	30	22	49
MM-015 [50]	MPR-R	152	77	77	31	NR; 70% at 36 mo
	MPR	153	68	68	14	NR; 62% at 36 mo
	MP	154	50	50	13	NR; 66% at 36 mo
ECOG E1A06 [51]	MPT-T	154	75	25	21	52.6
	MPR-R	152	70,4	32	19	47.7
MAIA [52]	Rd	369	82	57	34	NR
	Dara-Rd	368	93	81	NR; 60% at 48 mo	NR

Abbreviations: CR: Complete response; Dara-Rd: Daratumumab, lenalidomide, dexamethasone; Dara-VTD: Daratumumab, bortezomib, thalidomide, dexamethasone; Elo-Rd: Elotuzumab, lenalidomide, dexamethasone; KRd: Carfilzomib, lenalidomide, dexamethasone; mo: Months; MP: Melphalan, prednisone; MPT: Melphalan, prednisone, thalidomide; MPT-T: Melphalan, prednisone, thalidomide, thalidomide-maintenance; MPR: Melphalan, prednisone, lenalidomide; MPR-R: Melphalan, prednisone, lenalidomide, lenalidomide-maintenance; n: Number; N/A: Not applicable; NR: Not reached; ORR: Overall response rate; OS: Overall survival; PFS: Progression free survival; RD: Lenalidomide, high dose dexamethasone; Rd: Lenalidomide, low dose dexamethasone; Rd (cont): Lenalidomide, dexamethasone (continuous); Rd18: Lenalidomide, dexamethasone (18 cycles); TD: Thalidomide, dexamethasone; VCD: Bortezomib, cyclophosphamide, dexamethasone; VGPR: Very good partial response; VMP: Bortezomib, melphalan, prednisone; VMPT-VT: Bortezomib, melphalan, thalidomide, prednisone, bortezomib, thalidomide-maintenance; VRd: Bortezomib, lenalidomide, dexamethasone; and VTD: Bortezomib, thalidomide, dexamethasone.

**Table 2:** Results of randomized studies in relapsed/refractory multiple myeloma.

Trial/Author	Regimen	Patients, *n*	ORR, %	≥VGPR, %	Median PFS, mo	Median OS, mo or %
Lenalidomide-based						
ASPIRE [90]	KRd	396	87.1	70	26	48.3
	Rd	396	66.7	40	17,6	40.4
TOURMALINE-MM1 [91]	Ixa-Rd	360	78	48	20.6	N/A
	Rd	362	72	39	14.7	N/A
POLLUX [92]	Dara-Rd	286	93	80.4	44.5	NR
	Rd	283	76.4	49.3	17.5	NR
ELOQUENT-2 [93]	Elo-Rd	321	79	36	18.5	43.7
	Rd	325	66	30	15	39.6
Pomalidomide-based						
NIMBUS [94]	Pd	302	31	7	4	12.7
	Dex	153	10	1	1.9	8.1
Baz et al. [95]	PCD	34	65	12	9.5	N/A
	Pd	36	39	14	4.4	16.8
OPTIMISMM [96]	PVd	281	82	52.7	11.2	NR
	Vd	278	50	18.3	7.1	NR
APOLLO [97]	Dara-Pd	151	69	51	12.4	NR
	Pd	153	46	20	7	NR
ICARIA-MM [98]	Isa-Pd	154	60	32	11.5	NR; 72% at 12 mo
	Pd	153	35	9	6.5	NR; 63% at 12 mo
ELOQUENT-3 [99]	Elo-Pd	60	53	20	10.3	NR
	Pd	57	26	9	4.7	NR

Abbreviations: Dara-Pd: Daratumumab, pomalidomide, dexamethasone; Dara-Rd: Daratumumab, lenalidomide, dexamethasone; Dex: Dexamethasone; Elo-Pd: Elotuzumab, pomalidomide, dexamethasone; Elo-Rd: Elotuzumab, lenalidomide, dexamethasone; Ixa-Rd: Ixazomib, lenalidomide, dexamethasone; Isa-Pd: Isatuximab, pomalidomide, dexamethasone; KRd: Carfilzomib, lenalidomide, dexamethasone; mo: Months; n: Number; N/A: Not applicable; NR: Not reached; ORR: Overall response rate; OS: Overall survival; PCD: Pomalidomide, cyclophosphamide, dexamethasone; Pd: Pomalidomide, dexamethasone; PFS: Progression free survival; PVd: Pomalidomide, bortezomib, dexamethasone; Rd: Lenalidomide, dexamethasone; Vd: Bortezomib, dexamethasone; and VGPR: Very good partial response.
